# Machine Learning Case Study: Patterns of Kidney Function Decline and Their Association With Clinical Outcomes Within 90 Days After the Initiation of Renal Dialysis

**DOI:** 10.1053/j.akdh.2022.11.006

**Published:** 2023-01

**Authors:** Harvey W. Kaufman, Catherine Wang, Yuedong Wang, Hao Han, Sheetal Chaudhuri, Len Usvyat, Carly Hahn Contino, Robert Kossmann, Michael A. Kraus

**Affiliations:** Quest Diagnostics, Secaucus, NJ; Statistics and Data Science, Dietrich College of Humanities and Social Sciences, Carnegie Mellon University, Pittsburgh, PA; Department of Statistics and Applied Probability, College of Letters and Science, University of California - Santa Barbara, Santa Barbara, CA; Fresenius Medical Care, Waltham, MA; Fresenius Medical Care, Waltham, MA; Fresenius Medical Care, Waltham, MA; Fresenius Medical Care, Waltham, MA; Fresenius Medical Care, Waltham, MA; Fresenius Medical Care, Waltham, MA

**Keywords:** Machine learning, Renal dialysis, Prediction model, Estimated glomerular filtration rate

## Abstract

A case study explores patterns of kidney function decline using unsupervised learning methods first and then associating patterns with clinical outcomes using supervised learning methods. Predicting short-term risk of hospitalization and death prior to renal dialysis initiation may help target high-risk patients for more aggressive management. This study combined clinical data from patients presenting for renal dialysis at Fresenius Medical Care with laboratory data from Quest Diagnostics to identify disease trajectory patterns associated with the 90-day risk of hospitalization and death after beginning renal dialysis. Patients were clustered into 4 groups with varying rates of estimated glomerular filtration rate (eGFR) decline during the 2-year period prior to dialysis. Overall rates of hospitalization and death were 24.9% (582/2341) and 4.6% (108/2341), respectively. Groups with the steepest declines had the highest rates of hospitalization and death within 90 days of dialysis initiation. The rate of eGFR decline is a valuable and readily available tool to stratify short-term (90 days) risk of hospitalization and death after the initiation of renal dialysis. More intense approaches are needed that apply models that identify high risks to potentially avert or reduce short-term hospitalization and death of patients with a severe and rapidly progressive chronic kidney disease.

Machine learning builds models based on sample data that can be applied to predict future outcomes. Tom Mitchell’s often quoted definition is, “A computer program is said to learn from experience.”^1^ Machine learning has vast applications in society and health care.^2^ Only recently have we had the scope and scale of health care data and the affordability to analyze large data sets for machine learning.^3^ Despite applications in other fields, progress has been limited in nephrology.^4,5^ There are 2 basic approaches in machine learning: unsupervised learning for clustering patterns in unlabeled data and supervised learning for establishing associations between predictors and outcomes. This article provides a case study of machine learning in nephrology. Patterns of kidney function decline were categorized using unsupervised learning methods with longitudinal data. We then establish the association between the resulting patterns with clinical outcomes within 90 days after the initiation of renal dialysis using Cox proportional hazard and logistic regression models. Treatment of end-stage renal disease (ESRD) is expensive, accounting for approximately 7% of Medicare expenditures,^6^ and dialysis patients have a 3-year survival of approximately 50%.^7^ Leading causes of death are cardiovascular followed by infections and nonvascular sudden cardiac deaths.^8,9^ Pre-existing conditions that influence mortality include diabetes, cardiovascular disease anemia, bone disease, and chronic inflammation.^10–12^ Hospitalization and hospital readmission rates account for a substantial portion of medical expenses of renal dialysis patients. A study of 2007–2012 claims found that 80% of expenditures for patients with ESRD were for inpatient care.^13^ Yet, few studies assessed the risk of hospitalization among patients with chronic kidney disease (CKD) or for incident renal dialysis patients.^14–16^ In addition, there are limited studies that incorporate the trajectory of renal disease progression^17–21^ and cluster analysis into mortality risk assessment.^22^

Models that identify dialysis patients at high risk of adverse outcomes could offer valuable information on prognosis to inform the use of more aggressive care and supportive therapies that could avert or reduce hospitalization and death.^23–27^ The application of models with short-term outcomes may allow for interventions that may be more impactful. In this study, we identified a large group of patients who initiated renal dialysis and had available laboratory and claims data, enabling an analysis of outcomes within 90 days after the renal dialysis initiation.

## METHODS

Patients who initiated renal dialysis at Fresenius Medical Care, January 1, 2015, through September 30, 2020, were identified and matched with clinical laboratory testing provided by Quest Diagnostics. Data for each patient were also matched with hospitalization claims and death records. Each patient file was tokenized with a new identifier, and all identifying information was removed prior to data analysis. Likely mismatched patients were excluded. The Western Institutional Review Board (WIRB)-Corpernicus Group deemed this study to be exempt from human subjects review because all data were analyzed without personally identifiable information.

Using similar inclusion criteria as those in the study by O’Hare and colleague,^28^ patients were included when all the following criteria were met: (1) 2 years of clinical laboratory data prior to dialysis; (2) a minimum of 10 serum creatinine measurements in these 2 years; (3) at least 1 serum creatinine measurement within 45 days of dialysis initiation; (4) a minimum of 2 serum creatinine measurements distributed over at least 2 different quarters during the 2-year period before dialysis initiation. Patients with the following conditions were excluded: (1) sudden estimated glomerular filtration rate (eGFR) increases more than 20 mL/min/1.73 m^2^, likely due to overmatching of different patient data or (2) mean eGFR > 30 mL/min/1.73 m^2^ within 45 days prior to dialysis. The revised Chronic Kidney Disease Epidemiology Collaboration (CKD-EPI) 2021 equation was applied for eGFR calculation in this analysis.^29^

### Statistical Analysis

A cubic spline was fitted to eGFRs from each patient^30^ followed by functional data clustering methods to learn patterns of eGFR trajectories prior to the initiation of renal dialysis.^31^ Two approaches were evaluated: K-mean and functional principal component analysis. The cluster patterns and conclusions based on these 2 approaches are similar. Thus, only the results from the K-mean method are presented.

Demographic and laboratory measurements were summarized as means and standard deviations for continuous variables and as percentages for categorical variables in each eGFR trajectory cluster. One-way analysis of variance and chi-squared tests were, respectively, used to compare the different trajectory groups for continuous and categorical variables.

We considered survival time and whether a patient was hospitalized within 90 days of the initiation of renal dialysis as an outcome. To study the association between eGFR trajectory clusters and the outcomes, a Kaplan-Meier plot for survival time and bar plots for hospitalization were constructed. In addition, the Cox proportional hazard model was fitted for mortality and logistic regression model for hospitalization. The following 3 nested models for both the Cox proportional hazard and logistic regressions were considered:

- Model 1: 4 clusters based on eGFR trajectories;
- Model 2: model 1 plus demographic variables including age, sex, and race/ethnicity;
- Model 3: model 2 plus the following clinical laboratory data variables: 25-hydroxyvitamin D, albumin, urinary albumin-to-creatinine ratio (uACR), hemoglobin, hemoglobin A1c, intact parathyroid hormone, phosphate (as phosphorus), potassium, and white blood cell count.

Analyses were conducted with R version 4.1.2 (code-name “Bird Hippie”; R Foundation for Statistical Computing, Vienna, Austria), additionally employing the packages assist, fdapace, mgcv, and survival.

## RESULTS

The analysis included 2341 patients of whom 42% were female, and the overall mean age (standard deviation) was 64.9 years (12.3) (Table 1). By race/ethnicity, 62.7% were White, non-Hispanic; 15.7% were Black, non-Hispanic; 15.0% were Hispanic; 5.5% were Asian American; and 1.1% were categorized as other. The results of clinical laboratory tests are displayed in Table 2.

**Figure 1.**
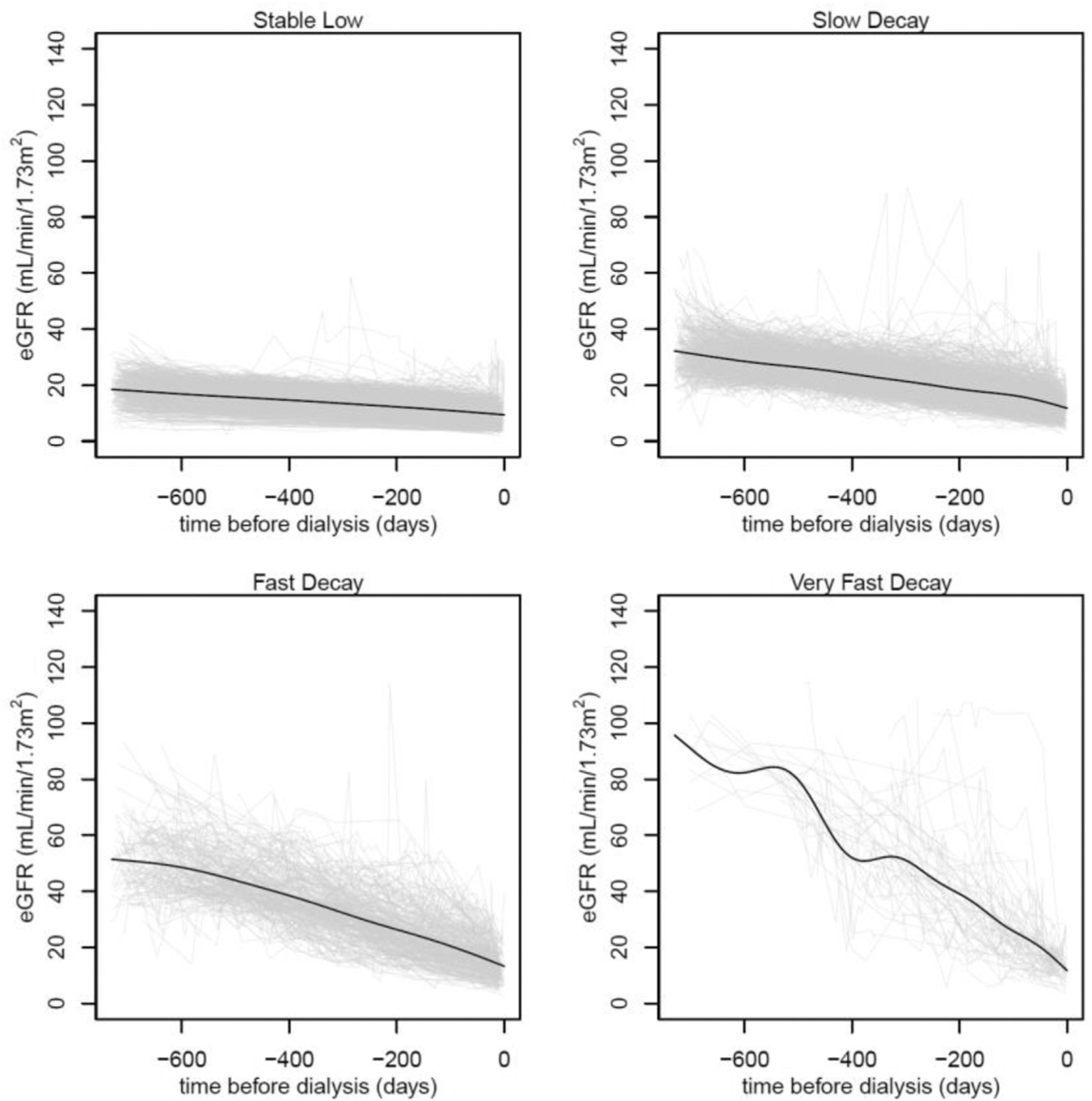
eGFR Values in the 2 years prior to the initiation of dialysis grouped by K-means eGFR trajectory clusters. The gray lines represent individual patient eGFR values, and the black lines represent the fitted cubic spline for each K-means eGFR trajectory cluster. Abbreviation: eGFR, estimated glomerular filtration rate. (For interpretation of the references to color in this figure legend, the reader is referred to the Web version of this article.)

The 2341 patients were grouped into 4 clusters of eGFR progression velocity by K-means: 1076 in the stable low cluster (minimal eGFR progression); 920 in the slow decay cluster (steady eGFR); 285 in the fast decay cluster (moderate eGFR progression); and 60 in the very fast decay cluster (fast eGFR progression). Figure 1 shows eGFR levels in the 2 years prior to the initiation of dialysis by cluster. Leading up to dialysis initiation, the decreases in eGFR were steepest for the very fast decay cluster, followed sequentially by fast, slow, and stable low decay clusters. The mean (standard deviation) time from eGFR of 20 mL/minute/1.73 m^2^ to dialysis was 669.5 days (94.8) for the stable low, 271.2 days (155.5) for slow decay, 121.4 days (93.1) for fast decay, and 75.2 days (61.8) for very fast decay clusters. Furthermore, faster decay groups had higher percentages of patients with an eGFR greater than 15 mL/minute/1.73 m^2^ at the time of dialysis initiation (Table 3).

Comparing demographic information, faster decay groups tended to be younger, had a higher proportion of male patients, and had a higher proportion of Hispanic individuals (Table 1). Comparing clinical laboratory test measures, faster decay groups tended to have lower levels of 25-hydroxyvitamin D, intact parathyroid hormone, and albumin; higher uACR; and slightly higher levels of hemoglobin A1C. The very fast decay cluster had higher white blood cell counts (Table 2). The 90-day mortality rates by clusters were 0.034 (37/1076) (stable low), 0.043 (40/920) (slow decay), 0.084 (24/285) (fast decay), and 0.117 (7/60) (very fast decay). The 90-day hospitalization rates by cluster were 0.219 (236/1076) (stable low), 0.252 (232/920) (slow decay), 0.295 (84/285) (fast decay), and 0.500 (30/60) (very fast decay).

**Figure 2.**
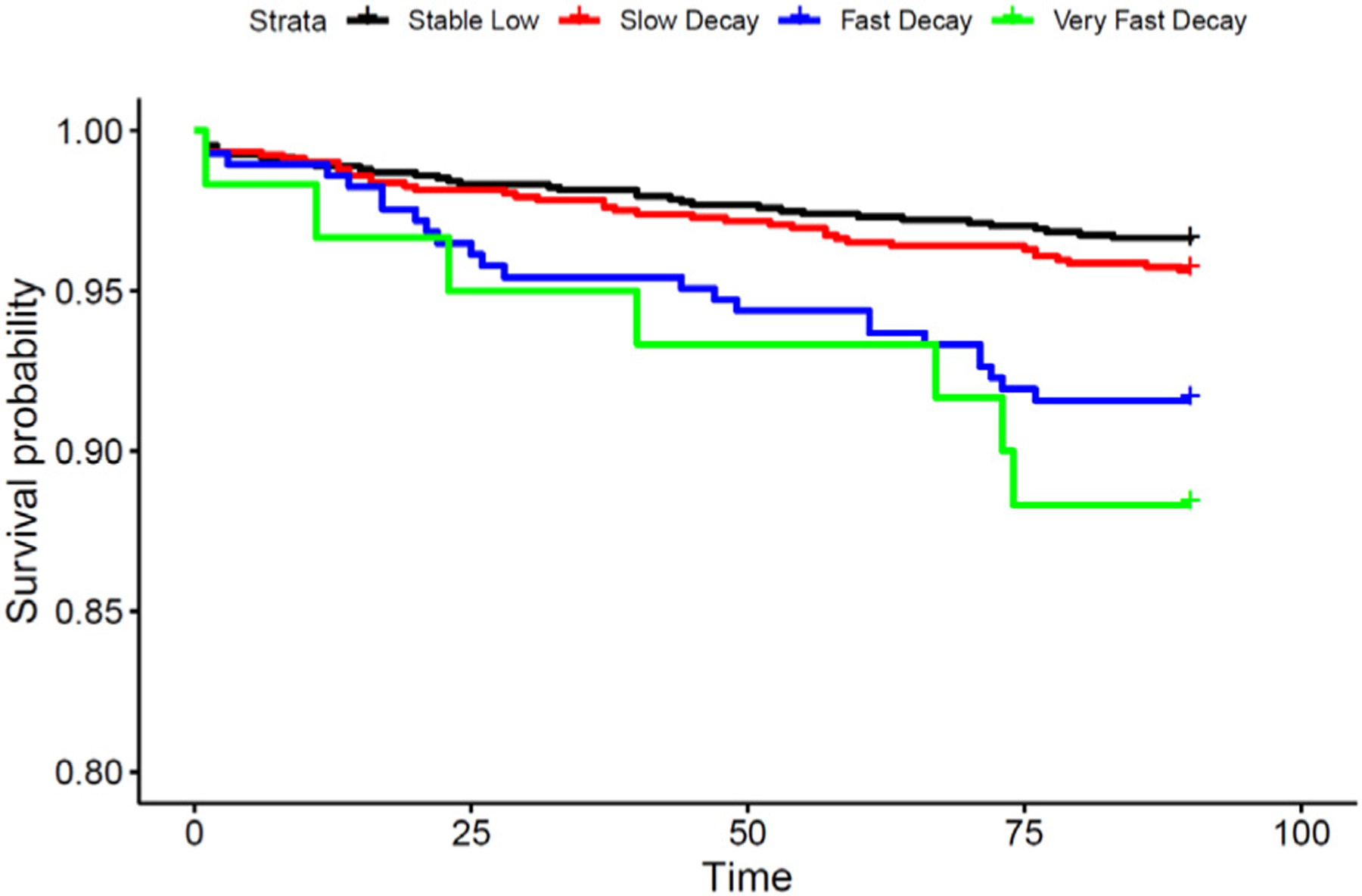
Kaplan-Meier estimate of the survival curve for each of the 4 eGFR trajectory clusters from K-means.

Faster decay of the renal function (the fast and very fast decay clusters) was associated with a higher risk of mortality (Fig 2) and hospitalization in the first 90 days after the initiation of dialysis. The estimated hazard ratio (95% confidence interval) in model 2 for mortality compared to the stable low cluster was 2.98 (1.78, 5.01) for the fast decay cluster and 4.53 (2.01, 10.19) for the fast decay clusters (Table 4). The estimated odds ratio (95% confidence interval) in model 2 for hospitalization compared to the stable low cluster was 1.57 (1.16, 2.10) for the fast-decay and 3.84 (2.24, 6.59) and for the very fast decay clusters (Table 5).

Cluster category was significantly associated with mortality and hospitalization, even after adjusting for demographic variables (Table 4: model 1 and model 2; Table 5: model 1 and model 2). However, the association was no longer significant after adding clinical lab variables (Table 4: model 3; Table 5: model 3).

## DISCUSSION

Machine learning can complement the efforts by nephrologists to improve health care delivery and prioritize limited health care resources. The cluster analysis described here provided the ability to differentiate the short-term risk of hospitalization and death among incipient renal dialysis patients by rate of decline in renal function as assessed by eGFR. Application of such categorization may provide clinicians an improved approach to prioritize resources and allocate more intense resources to patients at the highest risk of short-term adverse events (hospitalization and death).

Predialysis nephrology referral is recommended in the National Kidney Foundation Kidney Disease Outcomes Quality initiative, specifically for individuals with an eGFR <30 mL/minute/1.73 m^2^.^32,33^ Furthermore, approaches that can identify patients who are at the highest risk of hospitalization and risk mortality may provide additional emphasis for these patients to receive timely nephrology referral and appropriate care prior to the initiation of renal dialysis. Absent or late nephrology care prior to renal dialysis is associated with poorer outcomes,^34,35^ including mortality.^29,36–40^ Two Veterans Administration studies demonstrated the benefit of predialysis nephrology referral, specifically in lowering mortality.^41,42^ Approaches that increase nephrology referral prior to the initiation of renal dialysis should be clinically beneficial and may be cost-effective. Furthermore, understanding the rate of decline in eGFR when the eGFR is 15–20 mL/minute/1.73 m^2^ stratifies patients who may most benefit from short-term access to nephrology care.

Studies have found eGFR trajectories to predict mortality.^43,44^ Using cluster analysis, Santos and colleagues found that, among elderly patients, those with rapid eGFR declines compared to those patients with slower eGFR declines were more likely to have diabetes, cognitive impairment, and hospitalization prior to renal dialysis.^17^ Patients with rapid eGFR declines were less likely to have received nephrology care prior to renal dialysis and had higher mortality at 1 and 4 years after the initiation of dialysis. O’Hare and colleagues who evaluated patients prior to ESRD found an association between eGFR and mortality risk, but this was weaker among patients aged 65 years or older.^22^ These investigators suggested that mortality risk stratification should consider patient age. O’Hare and colleagues also found uACR was independently associated with mortality at all levels of eGFR in older adults with diabetes.^45^

Several trends among the 4 clusters warrant comment. The average age decreased with cluster progression, from 66 years in cluster 1 to 55.6 years in cluster 4. This may suggest that younger patients may have underlying conditions that contribute to faster CKD progression. The mean eGFR was lowest in cluster 1 (13.3 mL/minute/1.73 m^2^) and increased progressively through cluster 4 (33.8 mL/minute/1.73 m^2^). Patients in cluster 1, on average, already had severe CKD. In contrast, patients with the most rapid declines in renal function (cluster 4) started at higher levels of eGFR. The relationship with cluster 1 also having the highest mean age may suggest these patients may have had more years to reach this severe level of renal function. Other studies found a similarly higher portion of younger patients with more rapid declines in renal functiion.^18,19,23^ Older patients who survive long enough to reach more advanced stages of CKD are less likely than their younger counterparts to experience fast eGFR declines.

The mean uACR levels rose progressively from cluster 1 (16 mg/gram creatinine) to cluster 4 (36 mg/gram creatinine). This pattern is consistent with uACR as a measure of more rapid kidney functional impairment. Mean 25-hydroxyvitamin D levels were lower in clusters 3 and 4 than those in clusters 1 and 2. Studies have shown that low 25-hydroxyvitamin D levels in patients with ESRD are associated with a higher risk of all-cause mortality and a faster progression of kidney disease.^46–48^ It may be that even though patients in clusters 3 and 4 have higher initial eGFR levels than patients in clusters 1 and 2, the ability to synthesize vitamin D has already been impaired, and other measures better reflect impairment of renal function. The mean intact parathyroid hormone levels decreased progressively from cluster 1 (227 pg/mL) to cluster 4 (133 pg/mL) in our study. The expected relationship between 25-hydroxyvitamin D and intact parathyroid hormone was inversed. In multivariate analysis, parathyroid hormone levels were not associated with ESRD mortality.^49^ This may be why the measurement of 1,25-dihydroxyvitamin D is a better measure of vitamin D status among patients with an advanced renal disease.

Of note, this is the first study examining the risk of hospitalization and death among renal dialysis patients using the revised CKD-EPI 2021 calculation for eGFR^29^ recently adopted/promoted to mitigate applying the social construct of race to determination of eGFR. In addition, this study confirms the previous findings of O’Hare^17^ but does so with the revised CKD-EPI equation, with 42% of the study cohort being female; this is an important addition as only 1.6% of the prior study cohort were female. Together these 2 studies strongly confirm the relationship of rate of decline of eGFR and risk of death in the early time after the initiation of the renal replacement therapy. Our study also shows a strong relationship between hospitalization in the first 90 days and rate of decline of eGFR prior to the initiation of renal dialysis.

Limitations include that this study was based on patients who sought renal dialysis care through Fresenius Medical Care and who also had a minimum of 2 years of prior clinical laboratory testing through Quest Diagnostics. Claims data and record of hospitalization and death may be incomplete. Some patients who enter renal dialysis have had minimal prior health care services and may display different disease trajectories and outcomes from what is represented in this study. Finally, these observations should be validated in independent patient populations.

In summary, a clustering of eGFR trajectories, using unsupervised learning, was employed to demonstrate that patients within different clusters can be assigned risk severity of 90-day hospitalization and death. Earlier or more intense interventions may be useful to mitigate adverse outcomes such as hospitalization and death in patients with fast or very fast eGFR trajectories compared to patients with stable or slow declines in eGFR.

## Figures and Tables

**Table 1.** Demographic Information by eGFR Trajectory Clusters

Variable	All (*n* = 2341)	Stable Low (*n* = 1076)	Slow Decay (*n* = 920)	Fast Decay (*n* = 285)	Very Fast Decay (*n* = 60)	*P* Value
Age (mean years and standard deviation)	64.9 (12.3)	66.0 (11.9)	65.3 (11.8)	61.3 (13.2)	55.7 (16.7)	<0.001
Female (percent)	42.0	49.2	36.4	33.0	41.7	<0.001
Hispanic (percent)	15.0	12.6	16.2	18.9	20.0	0.039
White, non-Hispanic (percent)	62.7	61.5	64.1	62.6	64.4	0.751
Black, non-Hispanic (percent)	15.7	20.2	11.5	13.1	11.1	<0.001
Asian American (percent)	5.5	4.7	7.1	4.1	2.2	0.097
Other (percent)	1.1	1.1	1.1	1.4	2.2	0.894

Abbreviation: eGFR, estimated glomerular filtration rate.

Other race includes Native Hawaiian or other Pacific Islander or American Indian or Alaska Native. The *P* value for continuous variables (age) results from an analysis of variance of the variable across clusters. The *P* value for categorical variables results from a chi-squared test that assesses if counts across clusters are equal. Calculations are based on known demographic information (removes missing values).

**Table 2.** Clinical Laboratory Tests, by eGFR Trajectory Cluster

Variable	All (*n* = 2341)	Stable Low (*n* = 1076)	Slow Decay (*n* = 920)	Fast Decay (*n* = 285)	Very Fast Decay (*n* = 60)	*P* Value	Units
25-Hydroxyvitamin D	29.8 (12.5)	30.9 (12.2)	29.8 (12.5)	26.0 (12.8)	23.3 (14.3)	<0.000	ng/mL
Albumin	3.67 (0.43)	3.81 (0.35)	3.63 (0.39)	3.42 (0.48)	3.03 (0.66)	<0.001	g/dL
Albumin-to-creatinine ratio, random urine with creatinine	2071 (1870)	1639 (1397)	2204 (1845)	2731 (2537)	3551 (3086)	<0.001	mg/g creatinine
Hemoglobin	10.5 (1.3)	10.3 (1.2)	10.6 (1.3)	10.5 (1.6)	9.9 (1.4)	<0.001	g/dL
Hemoglobin A1C	6.80 (1.43)	6.54 (1.35)	6.89 (1.30)	7.32 (1.82)	6.93 (1.80)	<0.001	Percent
Intact parathyroid hormone	191.2 (158.2)	227.0 (184.5)	163.8 (120.8)	138.9 (99.9)	133.3 (180.8)	<0.001	pg/mL
Phosphorus as phosphate	4.6 (0.8)	4.8 (0.8)	4.5 (0.7)	4.6 (1.0)	4.6 (1.0)	<0.001	mg/dL
Potassium	4.6 (0.5)	4.6 (0.5)	4.6 (0.5)	4.5 (0.5)	4.5 (0.4)	<0.001	mmol/L
White blood cell count	7.4 (2.3)	7.3 (2.3)	7.4 (2.3)	7.2 (2.4)	8.3 (3.1)	0.008	1000/μL

Abbreviations: μL, microliter; dL, deciliter; eGFR, estimated glomerular filtration rate; g, gram; L, liter; mg, milligram; mL, milliliter; mmol, millimole; ng, nanogram; pg, picogram.

Individual patient laboratory tests are defined to be the average laboratory test value over the 2 years prior to the initiation of dialysis. Values in the table show the average (standard deviation).

**Table 3.** K-Means eGFR Trajectory Clustering Results

Variable	All (*n* = 2341)	Stable Low (*n* = 1076)	Slow Decay (*n* = 920)	Fast Decay (*n* = 285)	Very Fast Decay (*n* = 60)	*P* Value
eGFR mean (standard deviation) (mL/minute/1.73 m^2^)	18.6 (7.5)	13.3 (3.1)	20.6 (4.1)	28.6 (7.8)	33.9 (13.3)	<0.001
eGFR slope (SD) ([mL/minute/1.73 m^2^]/y)	−10.2 (10.7)	−4.8 (2.8)	−10.0 (4.8)	−22.0 (8.7)	−54.9 (21.9)	<0.001
baseline percent eGFR ≥ 15 (mL/minute/1.73 m^2^)	17.0	4.9	24.2	35.8	33.3	<0.001
Time since eGFR is 20 mL/minute/1.73 m^2^ until dialysis (standard deviation) (d)	431.0 (257.0)	669.5 (94.8)	271.2 (155.5)	121.4 (93.1)	75.2 (61.8)	<0.001

Abbreviation: eGFR, estimated glomerular filtration rate.

The mean eGFR is calculated for each individual during the 2 years prior to the initiation of dialysis. The eGFR slope ([milliliters/minute/1.73 m^2^]/year) is the estimated slope in the 2 years prior to the initiation of dialysis. The baseline eGFR ≤15 mL/minute/1.73 m^2^ variable indicates if the estimated (from the fitted spline) eGFR at dialysis start is ≥15 mL/minute/1.73 m^2^ or not. The time since eGFR is 20 mL/minute/1.73 m^2^ until dialysis is the time since the spline estimated eGFR is 20 mL/minute/1.73 m^2^ until the initiation of dialysis. The table shows the mean eGFR (standard deviation), mean eGFR slope (standard deviation), percent of individuals with baseline eGFR >15 mL/minute/1.73 m^2^, and mean time since eGFR is estimated to be 20 mL/minute/1.73 m^2^ to dialysis (standard deviation) by cluster. The *P* value for continuous variables (eGFR mean, eGFR slope, time since eGFR is 20 mL/minute/1.73 m^2^) results from an analysis of variance of the variable across clusters. The *P* values for categorical variables (baseline ≥ eGFR 15 mL/minute/1.73 m^2^) results from a chi-squared test which assesses if counts across clusters are equal.

**Table 4.** Hazard Ratio Estimates for Death in the First 90 d From Cox Proportional Hazard Models 1–3 Comparing the 4 eGFR Trajectory Clusters From K-Means

	Model 1 (Clusters)		Model 2 (Clusters + Demographic)		Model 3 (Clusters + Demographic + Clinical Laboratory Tests)	
Cluster	HR [95% CI]	*P* Value	HR [95% CI]	*P* Value	HR [95% CI]	*P* Value
Slow	1.270 [0.812, 1.985]	0.2953	1.310 [0.836, 2.053]	0.2384	0.902 [0.366, 2.219]	0.8217
Fast	2.506 [1.500, 4.198]	0.0005	2.983 [1.775, 5.014]	<10^−4^	1.290 [0.364, 4.568]	0.6931
Very fast	3.518 [1.569, 7.892]	0.0023	4.523 [2.008, 10.191]	0.0003	0 [0, Inf]	0.9973

Abbreviations: CI, confidence interval; eGFR, estimated glomerular filtration rate; HR, hazard ratio.

**Table 5.** Odds Ratio Estimates for Hospitalization in the First 90 d From Logistic Regression Models 1–3 Comparing the 4 eGFR Trajectory Clusters From K-Means

	Model 1 (Clusters)		Model 2 (Clusters + Demographic)		Model 3 (Clusters + Demographic + Clinical Laboratory Tests)	
Cluster	OR [95% CI]	*P* Value	OR [95% CI]	*P* Value	OR [95% CI]	*P* Value
Slow	1.200 [0.975, 1.477]	0.0845	1.231 [0.998, 1.520]	0.0527	2.409 [0.061, 93.015]	0.6374
Fast	1.487 [1.106, 1.988]	0.0079	1.566 [1.159, 2.105]	0.0032	1.042 [0.662, 1.643]	0.8580
Very fast	3.559 [2.098, 6.040]	<10^−4^	3.839 [2.240, 6.586]	<0.0001	0.806 [0.371, 1.669]	0.5723

Abbreviations: CI, confidence interval; eGFR, estimated glomerular filtration rate; OR, odds ratio.

## CLINICAL SUMMARY

- Risk stratification through unsupervised learning is useful to predict short-term (90 day) post-initiation renal dialysis risk of hospitalization and death.
- More aggressive interventions of patients categorized as fast or very fast declines in renal function are suggested to provide more timely and effective care and thereby reduce the risk of subsequent hospitalization and death.
